# Treat or observe? Managing Mycobacterium Chimaera in a patient with complex morbidties

**DOI:** 10.51894/001c.164573

**Published:** 2026-07-31

**Authors:** Fnu Bawna, Khabbab Alnimer, Abdallah Almawazreh, Malitha Hettiarachchi

**Affiliations:** 1 DMC - Sinai Grace

## 64

### Introduction

Mycobacterium chimaera, a non-tuberculous mycobacterium (NTM), is an emerging pathogen that presents diagnostic and therapeutic challenges, particularly in patients with underlying pulmonary disease and complex medical histories. Distinguishing true infection from colonization is often difficult when alternative explanations for respiratory symptoms are present.

### Case Description

A 61-year-old man with a history of follicular lymphoma transformed to diffuse large B-cell lymphoma, previously treated with chemotherapy, radiation, and autologous bone marrow transplantation, and a recent diagnosis of small cell lung cancer, presented with progressive dyspnea. His symptoms were attributed to multiple etiologies, including durvalumab-related pneumonitis treated with corticosteroids, RSV infection with superimposed bacterial pneumonia, pleural effusion requiring chest tube drainage, and heart failure with reduced ejection fraction. During the initial hospitalization, he received multiple courses of intravenous antibiotics and supportive care. Bronchoalveloar lavage was performed to evaluate infectious causes, and the patient was discharged while results were pending. He was readmitted shortly after discharge with worsening dyspnea, and the BAL subsequently returned positive for Mycobacterium chimaera. Infectious disease consultation recommended against initiating therapy due to the absence of radiographic findings typical of NTM pulmonary disease, such as cavitation or nodular infiltrates, and the presence of multiple alternative explanations for his respiratory symptoms. Treatment for M. chimaera requires prolonged multidrug therapy, typically for at least 12 months after culture clearance, and carries significant risk of adverse effects that were unlikely to improve the patient’s pulmonary status. Oncology and pulmonary teams discussed repeat bronchoscopy and BAL for further evaluation, with acknowledgment of possible need for intubation post-procedure. Palliative care was consulted to assist with goals-of-care discussions. Following multidisciplinary discussions with the care teams and family, the patient transitioned to hospice care.

### Discussion

Isolation of M. chimaera does not necessarily indicate active infection, as diagnosis of NTM pulmonary disease requires compatible clinical symptoms and characteristic radiographic abnormalities. In the absence of such findings, positive cultures may represent colonization. This case highlights the importance of integrating clinical, microbiologic, and radiographic data and emphasizes the role of multidisciplinary decision-making to avoid unnecessary prolonged therapy in patients with complex comorbidities and limited prognosis.

